# Protocol for using treeLFA to infer multimorbidity patterns in the form of disease topics from diagnosis data in biobanks

**DOI:** 10.1016/j.xpro.2025.104033

**Published:** 2025-09-07

**Authors:** Yidong Zhang, Xilin Jiang, Gil McVean, Gerton Lunter

**Affiliations:** 1 Department of Radiation Oncology, Peking Union Medical College Hospital, Chinese Academy of Medical Sciences and Peking Union Medical College, Beijing 100006, China; 2 Chinese Academy of Medical Sciences Oxford Institute, Nuffield Department of Medicine, University of Oxford, Oxford OX3 7BN, UK; 3 Big Data Institute, Li Ka Shing Centre for Health Information and Discovery, University of Oxford, Oxford OX3 7LF, UK; 4 British Heart Foundation Cardiovascular Epidemiology Unit, Department of Public Health and Primary Care, University of Cambridge, Cambridge CB2 0BB, UK; 5 Victor Phillip Dahdaleh Heart and Lung Research Institute, University of Cambridge, Cambridge CB2 0SR, UK; 6 Department of Epidemiology, Harvard T.H. Chan School of Public Health, Boston, MA 02115, USA; 7 Ellison Institute of Technology Oxford, Oxford OX4 4GE, UK; 8 Department of Epidemiology, University Medical Center Groningen, University of Groningen, Groningen 9700 RB, the Netherlands

**Keywords:** Bioinformatics, Genetics, Systems biology, computer sciences

## Abstract

Research on multimorbidity patterns promotes our understanding of the common pathological mechanisms that underlie co-occurring diseases. Here, we present a protocol to infer multimorbidity clusters in the form of disease topics from large-scale diagnosis data using treeLFA, a topic model based on the Bayesian binary non-negative matrix factorization. We describe steps for installing software, preparing input data, and training the model. We then detail post-processing procedures to obtain summarized results for downstream analyses.

For complete details on the use and execution of this protocol, please refer to Zhang et al.^1^

## Highlights

• Steps for using treeLFA to infer multimorbidity clusters are described
• Procedures for implementing treeLFA on an example dataset
• Guidelines for post-processing the inference result from treeLFA are presented
• Strategy of model selection for treeLFA is outlined

## Abstract (editor highlights)

Publisher’s note: Undertaking any experimental protocol requires adherence to local institutional guidelines for laboratory safety and ethics.

## Graphical abstract

**Figure:**
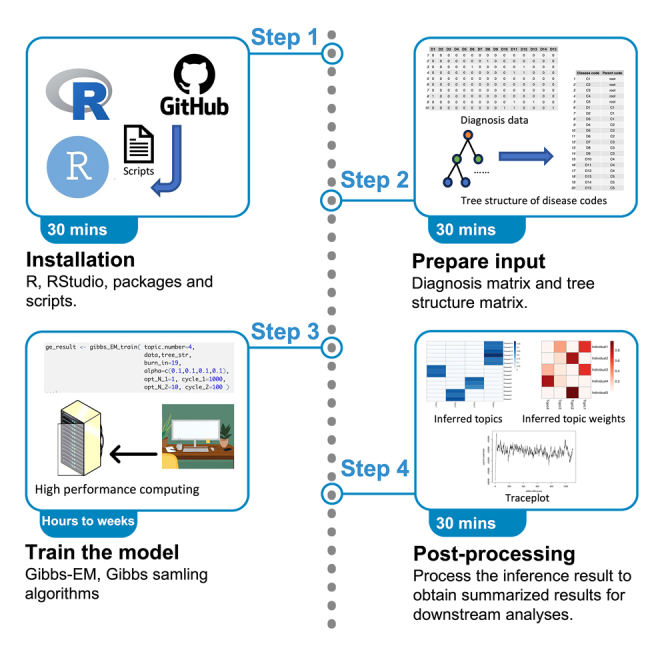

## Before you begin

**Figure 1:**
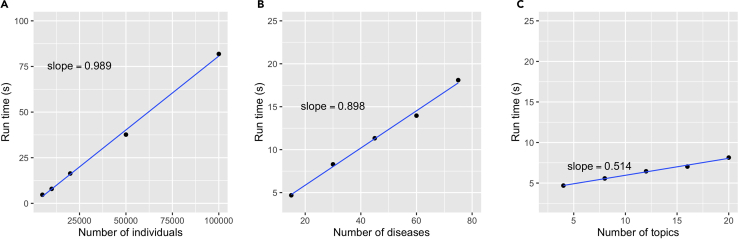
The computational time of Gibbs-EM training with different values set for three variables of the model (number of individuals, number of disease codes, and number of topics inferred) The slope of the fitted line was calculated by scaling the largest value of runtime to be the same as the largest value of the independent variables (x-axis). The full result is in Table 1. (A) The relationship between the computational time and the number of individuals in the training dataset. (B) The relationship between the computational time and the total number of diseases to be analysed. (C) The relationship between the computational time and the number of topics to be inferred.

Multimorbidity refers to diseases that tend to co-occur within the same individuals. With the increasing incidence of multimorbidity and their negative impacts on patients’ health, it is imperative to study them and their etiology to guide the development of new prevention and treatment strategy.^2^ In recent years the establishment of biobanks worldwide has enabled systematic study of multimorbidity patterns.^3^ “treeLFA” (latent factor allocation with a tree-structured prior) models the diagnosis data of patients by viewing individuals as “documents” and diseases as “words” in the topic modeling setting, and “topics” of diseases are probability vectors of diseases, where diseases that are likely to co-occur will have large probabilities in the same topic, while other diseases in the topic have negligible probabilities. For details of the structure and generative process of the model, refer to Figure 1 and the “treeLFA” subsection in the “Method Details” section in the primary publication. Compared to other topic models like LDA (Latent Dirichlet Allocation),^4^ treeLFA can more accurately infer disease topics. In addition, it brings additional power for the downstream GWAS using peoples’ inferred topic weights as traits, to find genomic loci that are associated with a group of diseases inclined to co-occur.

This protocol details the steps to infer multimorbidity clusters in the form of disease topics as well as individuals’ corresponding topic weights, stating from a diagnosis input data and a two columns table encoding a hierarchical structure of disease codes describing the relationship of all diseases in general (such as the ICD-10 disease coding system). The algorithm can also work without a specific tree structure of diseases, in which case no prior similarity structure are assumed among diseases.

To apply treeLFA, the only prerequisite is to install the R software and download a few R packages and scripts from the Github repository for treeLFA. It is recommended to train treeLFA model on a high-performance computing (HPC) facility if a biobank-scale dataset (for example, hundreds of diseases, hundreds of thousands of individuals) is used.

**Table 1:** Relationship between the training time and parameters of the treeLFA model/the computing equipment on simulated and UK biobank datasets

People	Disease	Topic	Cycle1^a^	Cycle2^a^	opt_N_1^a^	opt_N_2^a^	Computing nodes	Equipment	Dataset	Parallel tasks	Memory	Runtime (s)
1000	15	4	50	5	1	10	1	laptop	simulation	1	16G	1.65
5000	15	4	50	5	1	10	1	laptop	simulation	1	16G	4.69
10000	15	4	50	5	1	10	1	laptop	simulation	1	16G	7.92
20000	15	4	50	5	1	10	1	laptop	simulation	1	16G	16.35
50000	15	4	50	5	1	10	1	laptop	simulation	1	16G	37.66
100000	15	4	50	5	1	10	1	laptop	simulation	1	16G	81.86
5000	15	8	50	5	1	10	1	laptop	simulation	1	16G	5.57
5000	15	12	50	5	1	10	1	laptop	simulation	1	16G	6.45
5000	15	16	50	5	1	10	1	laptop	simulation	1	16G	7.03
5000	15	20	50	5	1	10	1	laptop	simulation	1	16G	8.14
5000	30	4	50	5	1	10	1	laptop	simulation	1	16G	8.29
5000	45	4	50	5	1	10	1	laptop	simulation	1	16G	11.33
5000	60	4	50	5	1	10	1	laptop	simulation	1	16G	13.96
5000	75	4	50	5	1	10	1	laptop	simulation	1	16G	18.1
100000	100	10	100	10	1	10	1	HPC	UKB	8	200G	1556.6
100000	100	10	100	10	1	10	1	HPC	UKB	1	200G	4101
100000	100	10	100	10	1	10	1	HPC	UKB	8	2G	1671
100000	100	10	100	10	1	10	1	HPC	UKB	1	2G	3922

a Cycle1, Cycle2, opt_N_1, opt_N_2 are the parameters of the Gibbs-EM training function (see step 3).

### Preparation 1: Find the appropriate computing facility

**Timing: Variable**

***Note:*** The computer memory requirement of training the treeLFA model is not high. The largest matrices stored during the inference are the input data matrix and the topic assignment matrix (both are “number of people” by “number of diseases” matrices). The size of a dataset containing hundreds of disease codes and half a million people is less than 1 GB (biobank scale data). Although the storage of a large number of posterior samples of hidden variables requires relatively large memory space (see step 5), the overall computer memory size requirement should be easily met if a HPC facility is available.

***Note:*** The training of treeLFA on biobank-scale data can take a long time (up to around two weeks). Figure 1 shows the increase of computational time as the number of people, diseases and inferred topics increases (each time one variable is varied with the other two fixed) on simulated data. The computational time scales linearly with the number of people and number of disease codes in the input dataset (scaling factor near 1, calculated by scaling the maximum of the dependent/independent variables to the same value) and the number of topics inferred (scaling factor near 0.5) (Figure 1). We also use the Gibbs-EM algorithm to infer 10 topics on a real UKB dataset containing 100 disease codes for 100,000 people on a HPC facility (use 48 cores on one computing node; CPU clock frequency: 2.4 GHz). On a single computing node, training with Gibbs-EM for 200 EM cycles took 4101 s, while parallel training (8 parallel tasks) on one node took 1556.6 s (Table 1). The size of computer memory didn’t significantly influence the computational time (Table 1). On the full UK biobank data (assume there are 500,000 people and 400 diseases, and 50 topics are inferred), a typical parallel training (8 parallel tasks, 2,000 EM cycles) with the Gibbs-EM algorithm is estimated to take around 9 days. The Gibbs sampling step usually take much less time than the Gibbs-EM training, as less total Gibbs sampling iterations are needed.

### Preparation 2: Install required software and source scripts of the algorithm

**Timing: 30 min**

1. Install R (version 4.2.2 or higher) and RStudio.

***Note:*** It is recommended to run the code in RStudio for the convenience of debugging, but the code can also be run in a console R session.

***Note:*** The scripts of treeLFA have been tested with the latest released R software (R 4.4.3).

2. Download R scripts for treeLFA from the Github repository:
  a. Navigate to the Github webpage for treeLFA: *https://github.com/zhangyd10/treeLFA-demo-CG*, click the green button “Code”, and then in the drop-down list click “Download ZIP”. The downloaded directory has the name “treeLFA-demo-CG-master.zip”.
  b. Extract all files from the downloaded zip file. The scripts for running treeLFA are in the “docs” folder in the directory.
3. Install and load R libraries: troubleshooting 1.

install.packages("data.table")

install.packages("Rcpp")

install.packages("RcppParallel")

install.packages("dqrng")

install.packages("pheatmap")

install.packages("RColorBrewer")

install.packages("coda")

install.packages("lsa")

install.packages("ggplot2")

install.packages("BiocManager")

library(BiocManager)

BiocManager::install("scran")

library(data.table)

library(Rcpp)

library(RcppParallel)

library(dqrng)

library(pheatmap)

library(RColorBrewer)

library(coda)

library(lsa)

library(scran)

library(ggplot2)

***Note:*** The “scran” package needs to be installed with the “BiocManager” package, and the installation can take a few minutes, as the package has many dependencies. When asked by the R software, users can choose not to update the relevant packages to save time.

***Note:*** The list of all the R packages attached/loaded after loading the required packages for treeLFA is shown below (output by the “sessionInfo” function):

Base packages attached: stats4,stats,graphics_,grDevices_,utils_,datasets_,methods;

Other attached packages: ggplot2_3.5.1,scran_1.34.0,ggplot2_3.5.1,scran_1.34.0,scuttle_1.16.0,SingleCellExperiment_1.28.1,SummarizedExperiment_1.36.0,Biobase_2.66.0,GenomicRanges_1.58.0,GenomeInfoDb_1.42.3,IRanges_2.40.1,S4Vectors_0.44.0,BiocGenerics_0.52.0,MatrixGenerics_1.18.1,matrixStats_1.5.0,coda_0.19–4.1,RColorBrewer_1.13,pheatmap_1.0.12,dqrng_0.4.1,RcppParallel_5.1.10,Rcpp_1.0.14,data.table_1.16.4;

Packages loaded via a namespace:

gtable_0.3.6,xfun_0.5,lattice_0.226,generics_0.1.3,vctrs_0.6.5,tools_4.4.3,parallel_4.4.3,tibble_3.2.1,cluster_2.1.8,pkgconfig_2.0.3,BiocNeighbors_2.0.1,Matrix_1.72,lifecycle_1.0.4,GenomeInfoDbData_1.2.13,compiler_4.4.3,statmod_1.5.0,munsell_0.5.1,bluster_1.16.0,codetools_0.220,htmltools_0.5.8.1,yaml_2.3.10,pillar_1.10.1,crayon_1.5.3,BiocParallel_1.40.0,DelayedArray_0.32.0,limma_3.62.2,abind_1.48,tidyselect_1.2.1,metapod_1.14.0,locfit_1.59.12,rsvd_1.0.5,digest_0.6.37,dplyr_1.1.4,BiocSingular_1.22.0,fastmap_1.2.0,grid_4.4.3,colorspace_2.11,cli_3.6.3,SparseArray_1.6.2,magrittr_2.0.3,S4Arrays_1.6.0,withr_3.0.2,edgeR_4.4.2,scales_1.3.0,UCSC.utils_1.2.0,rmarkdown_2.29,XVector_0.46.0,httr_1.4.7,igraph_2.1.4,ScaledMatrix_1.14.0,beachmat_2.22.0,evaluate_1.0.3,knitr_1.49,irlba_2.3.5.1,rlang_1.1.5,glue_1.8.0,rstudioapi_0.17.1,jsonlite_1.8.9,R6_2.5.1,zlibbioc_1.52.0.

4. Source R scripts for treeLFA: troubleshooting 1.

setwd("/<full path to/treeLFA-demo-CG-master/docs/")

Rcpp::sourceCpp("treeLFA.ge.new.cpp")

Rcpp::sourceCpp("treeLFA.g.new.cpp")

Rcpp::sourceCpp("pl_mc.cpp")

source(file="treeLFA.functions.R")

***Note:*** Warning messages (not error messages) that appear while sourcing the scripts can be ignored.

## Key resources table

**Table:** 

REAGENT or RESOURCE	SOURCE	IDENTIFIER
**Deposited data**		

UK Biobank	Bycroft et al.^2^	https://biobank.ctsu.ox.ac.uk Data field: 41202, 41204, 22418
ICD-10 coding system	World Health Organization	https://icd.who.int/browse10/2016/en
Example input data	This paper	https://github.com/zhangyd10/treeLFA-demo-CG/tree/main/docs/simu.train.data.txt
Example tree structure	This paper	https://github.com/zhangyd10/treeLFA-demo-CG/tree/main/docs/tree.str.simu.txt

**Software and algorithms**		

treeLFA scripts	This paper	https://github.com/zhangyd10/treeLFA-demo-CG https://doi.org/10.5281/zenodo.8077452

## Step-by-step method details

### Prepare input for treeLFA

**Timing: 30 min**

In this step, prepare the two input files for training the treeLFA model.

***Note:*** There are two inputs for treeLFA: individuals’ diagnosis data, and the tree structure of disease codes. The diagnosis data is a binary matrix which records the presence (diagnosis) and absence of all disease codes for all individuals. The tree (hierarchical) structure of disease codes describes the relationship of all diseases, on which the informative prior for topics is constructed. The assumption of the prior is that disease codes close to each other on the tree structure are more likely to be active in the same topic compared to those that are far from each other.

***Note:*** There are example files of the two input files for treeLFA in the directory downloaded from Github.

1. Prepare the diagnosis data for individuals: troubleshooting 2.
  a. Read the small example input dataset into R using the “fread” function in the “data.table” package:

data <- fread("simu.train.data.new.txt")

**Figure 2:**
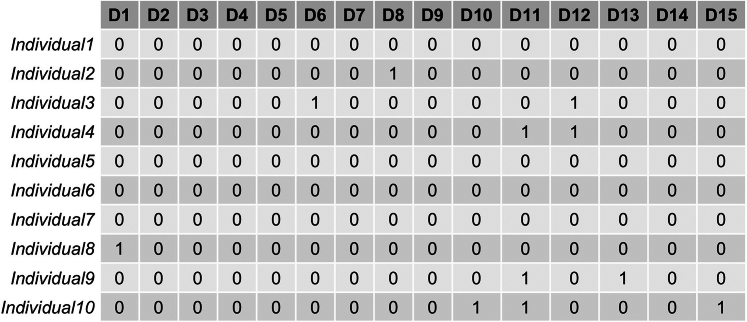
An example of the input diagnosis data matrix Each row is a patient, and each column is a disease code. 0 and 1 represent the absence and presence of a disease code diagnosed for a patient.

***Note:*** The diagnosis data should be prepared as a binary matrix that can be loaded into R (for instance, as a “.txt” file). Each row of the matrix corresponds to an individual, and each column corresponds to a disease code. Zeros and ones in the matrix represent the absence and presence of disease codes diagnosed for individuals, and only zeros and ones should appear in the matrix (Figure 2). The column names of the matrix should be the disease codes to be analyzed and appear exactly in the second input file describing the disease code hierarchy (see below).

***Note:*** The example training data in the downloaded package contains the diagnostic record of 15 disease codes for 5,000 individuals (Figure 2).

**Figure 3:**
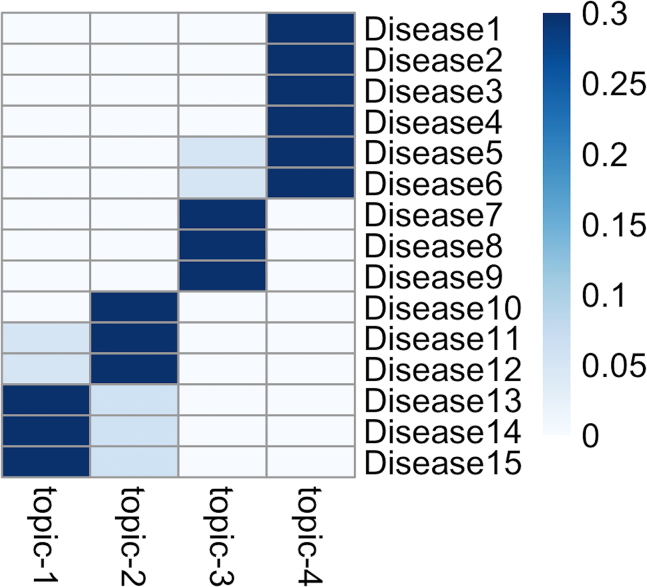
The four topics (“true” topics) used to simulate the example input diagnosis data for this protocol Each topic is a vector of 15 probabilities (for the 15 disease codes).

***Note:*** The example input diagnosis data for treeLFA is simulated using the generative process of treeLFA and four topics with simple structure. Figure 3 shows the four topics used for the simulation, which are vectors containing the probabilities of the 15 disease codes in sequence. In each topic, the probabilities of several diseases are larger than zero (for simplicity, all active disease codes in topics are assigned with a probability of 0.3, and all inactive disease codes are assigned with a zero probability), indicating these diseases tend to co-occur on the same individuals.

2. Prepare the input file which encodes the tree structure of disease codes: troubleshooting 3.
  a. Read the tree structure of disease codes for the example input dataset into R:

tree_str <- fread("tree.str.simu.txt")

**Figure 4:**
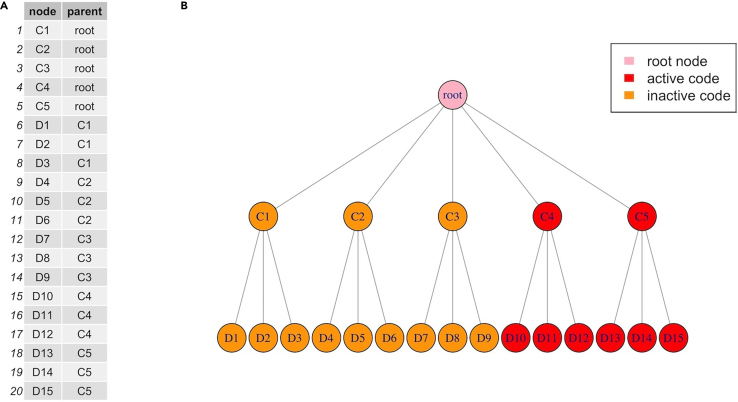
An example of the input matrix encoding the tree structure of disease codes and a schematic of the tree structure (A) The 1^st^ column of the tree structure matrix contains all the terminal (on the bottom layer of the tree) and internal disease codes on the tree structure, and the 2^nd^ column contains the parent codes of the corresponding disease codes in the first column. (B) The tree structure of disease codes encoded by the matrix in panel A. It has 3 layers, the 1^st^ layer contains only the root node, the 2^nd^ layer has five internal nodes (C1, C2, C3, C4 and C5), and each node in the 2^nd^ layer has 3 children nodes on the 3^rd^ layer (for instance, node C1 has children nodes D1, D2 and D3). The red nodes on the tree correspond to the active disease codes (disease codes with a probability larger than 0) in topic 4 in Figure 3.

**CRITICAL:** The tree structure of disease codes is encoded using a two-column matrix. The first column contains all the disease codes to be analyzed (terminal codes on the tree structure) as well as their parent codes (internal codes on the tree structure, except for the root code) on the tree structure. The second column specifies the parent codes of the corresponding disease codes in the first column. The root code (node) of the tree structure should be named as “root” and appears in the second column. Each disease code in the first column should have a single unique parent code in the second column (see an example of the input file in Figure 4A, and visualize the tree structure of the 15 disease codes in Figure 4B).

***Note:*** Terminal disease codes (disease codes appear in the first but not the second column of the matrix) in the tree structure matrix and the diagnosis data matrix (as column names) should have the same names. The algorithm will check this, and an error message will appear if this is not the case.

***Note:*** The schematic of the tree structure of disease codes (Figure 4B) can be generated using the “igraph” R package. The tree structure matrix (Figure 4A) can be directly converted to an igraph object using the “graph.edgelist” function, and then be plotted. The colors of nodes in Figure 4B (denoting active/inactive disease codes in a topic) are manually set.

***Note:*** If there isn’t a tree structure of disease codes to be used for the inference of disease topics, a tree structure matrix in which the “root” code is the parent code of all disease codes (“flat” tree structure) will be used by the algorithm by default (in this case, the tree structure file does not need be to passed to the inference function), which means all entries in the 2^nd^ column of the matrix are “root”. The influence of using this non-informative tree structure is introduced in the note below.

***Note:*** We found on both simulated and real-world data that the incorporation of a tree-structured prior for topics based on a disease classification system into the model can only slightly improve the inference accuracy. The input data is the major decisive factor of the inference result, which means the tree structure only plays an active role when there is limited input data (for instance, for diseases with low prevalence in the data or diseases whose comorbidity patterns are weak). One way to assess the influence of the tree structured prior is to train a model using a “flat” tree structure (as is introduced in the previous note), in which all diseases codes are parallelly placed under the root code. This is a non-informative tree structure, and the corresponding model was named “flatLFA” in the primary publication (Figures 2 and 9; “Validation of treeLFA and comparison with related topic models” and “Analyses on hundreds of ICD10 codes” subsections in the “Results” section).

***Note:*** For the preparation of real-world data, users can refer to the “inference on the top-100 UKB dataset” subsection in the “Method Details” section in the primary.

### Perform inference for the treeLFA model

**Timing: Variable (hours to weeks, depending on the size of the input data and the computing facility)**

Perform optimization of the hyperparameters and inference of the hidden variables of treeLFA at the same time using the Gibbs-EM algorithm, and then collect posterior samples of hidden variables using the Gibbs sampling algorithm.

***Note:*** In the following sections, the hidden variables and hyperparameters of treeLFA are present with bold fixed-width font, while other parameters of functions, R objects (including the result objects) as well as its elements and function names are present with italic fonts.

**Figure 5:**
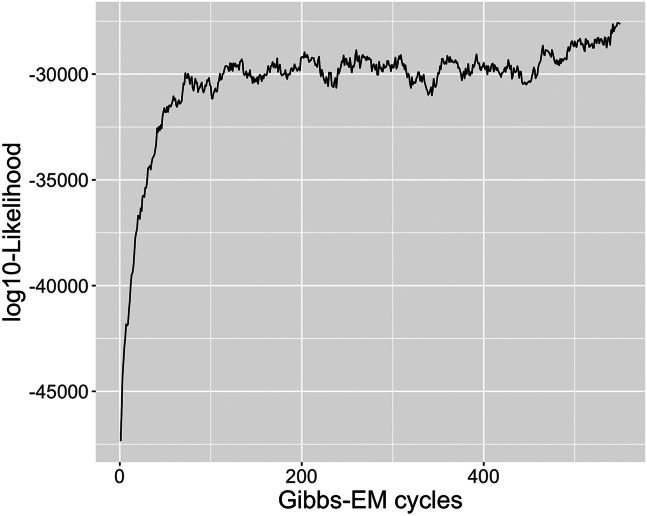
The traceplot of the likelihood of the treeLFA model on the example training dataset is used to monitor the convergence of the Gibbs-EM chain

3. Use the Gibbs-EM algorithm for treeLFA to optimize the Dirichlet prior (**“alpha”**) for individuals’ topic weight variables, and at the same time to infer all hidden variables. Troubleshooting 4.
  ge_result <- gibbs_EM_train( topic.number=10,
    data=data,
    tree_str=tree_str,
    alpha=rep(0.1,10),
    burn_in=19,
    opt_N_1=1, cycle_1=1000,
    opt_N_2=10, cycle_2=100 )
  **CRITICAL:** The parameters that need to be set for the Gibbs-EM training function (“*gibbs_EM_train*”) are shown in the example code above (all these parameters except for the “*tree_str*” parameter need to be set since there will be no default values set for them automatically by the algorithm):
  a. “*topic.number*”: the total number of topics to be inferred; (see the note below).
  b. “*data*”: the input diagnosis data matrix;
  c. “*tree_str*”: the matrix encoding the tree structure of disease codes;
  d. “*alpha*”: The initial values of entries in the hyperparameter vector **“alpha”** (the Dirichlet prior for individuals’ topic weight variables). In this example **“alpha”** is set to be a vector of 0.1, which is the “true” **“alpha”** vector used to simulate the input diagnosis data. On real world datasets, it is recommended to set **“alpha”** as (1,0.1,...0.1), in which the first entry corresponds to the “empty” (“healthy”) topic that will be usually inferred (see notes below);
  e. "*burn_in*": Denotes the number of Gibbs sampling iterations to be run in each E-step of the Gibbs-EM algorithm before the collection of one posterior sample of all hidden variables in this E-step. 19 is the recommended value for this argument;
  f. *“opt_N_1”, “cycle_1”, “opt_N_2”, “cycle_2”*: There are two stages of the Gibbs-EM training for the treeLFA model. “*cycle_1*” and “*cycle_2*” are the total numbers of EM cycles in the two stages, and “*opt_N_1*” and “*opt_N_2*” are the numbers of posterior samples of all hidden variables collected in each E-step (these posterior samples of hidden variables will be used in the M-step to optimize “**alpha**”). Note that “*opt_N_1*” is usually set to be one to speed up the training, and “*opt_N_2*” can be set to an integer larger than one (ten is recommended) to enable a more accurate optimization of “**alpha**”;
    ***Note:*** As in situations where real-world datasets are analyzed, here we do not have an idea about the correct number of topics to be inferred before we see the exploratory inference result, so we set a relatively large number of topics to infer (ten topics here, while the true number of topics is four) for the Gibbs-EM algorithm. As will be seen in the following steps, duplicated topics will be inferred as the result of an excessive number of topics set for the algorithm. In the post-processing steps, duplicated topics will be combined. If we set the number of topics to be four, we will have an accurate inference result for all topics directly (not shown in this protocol).
    ***Note:*** It is recommended to train treeLFA model with the Gibbs-EM algorithm on a HPC facility. Parallel computing can be employed by simply setting the number of parallel tasks (or computing nodes) to be more than 1 in the command script submitted to the task scheduler of the HPC, as the algorithms were coded using the R package “RcppParallel”.
    ***Note:*** To enable the parallel implementation of the inference algorithm for treeLFA using Rcpp and RcppParallel, we used multiple layers of random number generators in the Rcpp code. On the parallel inference workers the partition of the input data was used as one of the parameters controlling the generation of random numbers. As a result, setting a seed in the wrapper R script won’t guarantee the reproducibility of the inference result.
    ***Note:*** If users choose not to provide a tree structure of diseases, a non-informative tree structure will be used by default (see notes in step 2)
    ***Note:*** “**alpha**” is optimized in the M-step using a “fixed-point method” by maximizing the expectation of the log-likelihood of the data calculated in the E-step (section 4.3 of Data S1 of the primary publication).
    ***Note:*** The step-by-step mathematical derivation for the Gibbs-EM algorithm can be found in Data S1 of the primary publication.
    ***Note:*** The inference results are stored in the “*ge_result*” object, including values of all hidden variables and hyperparameter (“**alpha**”) given by the last Gibbs-EM iteration. Hidden variables of treeLFA in the result object include the probability variables of all disease codes in all topics (“**Phi**”), the indicator variables of disease codes in topics (“**I**”), the transition probability variables of the Markov process on the tree structure of diseases (“**rho**”), and the topic assignment variables for all disease codes of all individuals (“**Z**”).
    ***Note:*** The indicator variables (with 1 and 0 denoting active/inactive disease codes in topics) and the Markov process generating the indicator variables are used to increase the sparsity of topics. As are specified by the generative process of treeLFA, the probability of active/inactive disease codes in topics are sampled from different Beta priors, such that active diseases codes have large probability, while inactive disease codes have near-zero probability. In addition, the transition probabilities of the Markov process make it difficult to generate active indicators, resulting in most topics having only a limited numbers of active disease codes.
    ***Note:*** Most of the hidden variables of treeLFA are randomly initialized (“**Z**” and “**Phi**” are sampled from categorical and Beta distributions respectively) internally by the algorithm before the training starts. Some variables are initialized empirically: “**I**” are initialized as 0, and “**rho**” is initialized as (0.1, 0.5). The hyperparamters of the Beta prior distributions for “**Phi**” are empirically chosen and then fixed according to the prevalence of diseases in the input dataset. Beta(0.1, 500)/Beta(2, 4), Beta(0.1, 1000)/Beta(1.5, 3) and Beta(0.1, 4000)/Beta(1.2, 3) are used as the prior distribution of the probability of inactive/active disease codes in datasets where the minimal prevalence of disease codes is in the range of [0.01, 1), [0.005, 0.01) and(0, 0.005). These Beta distributions are selected such that the probability of inactive disease codes will be significantly smaller than the probability of active disease codes, and the probability of active disease codes in topics won’t be too small. The Beta priors for the two transition probability ($\rho_{01},\rho_{11}$) of the Markov process are Beta(3, 20) and Beta(3, 3), such that for one thing the probability of an inactive disease code having an active child code is small (small $\rho_{01}$ increases the sparsity of topics), and for another thing a large proportion of the children codes of an active parent code will still be active (relatively large $\rho_{11}$). During the development of the treeLFA model, we studied the influence of the initialization of hidden variables and hyperparameters, and confirmed that it has minimal influence on the final inference result.
    ***Optional:*** After training with the Gibbs-EM algorithm, use the traceplot of the model’s likelihood to monitor the convergence of “Gibbs-EM” (Figure 5):
    plot( 1:length(ge_result$L_all),ge_result$L_all, type=“line”,
     xlab=“Gibbs-EM cycles”,ylab=“Log10-Likelihood” )
    ***Note:*** The “*L_all*” vector in the “*ge_result*” object records the likelihood of the treeLFA model for each Gibbs-EM iteration, and is plotted against the Gibbs-EM iterations to monitor the convergence of the Gibbs-EM chain (Figure 5). A traceplot with a sharp increase in likelihood at the beginning of the training, stabilizing at the ends of both training stages (with no long-term trends) and moving up and down freely denotes good convergence and mixing. Troubleshooting 6.
    ***Optional:*** Continue the training with Gibbs-EM algorithm using the inference results of previously stopped Gibbs-EM chains.
    ge_result <- gibbs_EM_train( topic.number=10,
      data=data,
      tree_str,
      Phi=ge_result$Phi_samples,
      I=ge_result$I_samples,
      rho=ge_result$rho_samples,
      Z=ge_result$Z_samples,
      alpha=ge_result$alpha_samples,
      burn_in=19,
      opt_N_1=1, cycle_1=1000,
      opt_N_2=10, cycle_2=100 )
    ***Note:*** On very large datasets, the full Gibbs-EM training can be divided into several phases, such that each phase will not take too long. By setting the arguments for hidden variables (“**Phi**”, “**I**”, “**rho**”, “**Z**”) and the hyperparameter “**alpha**” in the “*gibbs_EM_train*” function, previous training can be continued (values of hidden variables and hyperparameters in the result object given by previous training are used to initiate the hidden variables and hyperparameters for the new training phase).
    ***Note:*** There is no specific recommendation on the numbers of phases to divide the full training process into. It mainly depends on how long the users want each training phase to be, and the estimated training time of the full training process (see the “find the appropriate computing facility” subsection in the “before you begin” section)
    ***Note:*** As the training with the Gibbs-EM algorithm on large dataset can take a long time, the “*gibbs_EM_train*” function will periodically (every 50 cycles for the 1^st^ training phase and every 5 cycles for the 2^nd^ training phase) save intermediate inference result (the current values of the hidden variables and hyperparameters). In this way, if a training is interrupted for some reasons, users can continue the previous training using the intermediate result to initialize the hidden variables, instead of starting from scratch again. The intermediate result is named “*intermediate_result.RData*” and can be found in the “*doc*” directory in the downloaded directory. It has the same data structure as the *“ge_result”* object when loaded into the R session using the “*readRDS*” function.
4. Use Gibbs sampling to simulate posterior distributions of hidden variables with “**alpha**” fixed (given by the finished Gibbs-EM training) and hidden variables initialized with values from the last cycle of the finished Gibbs-EM training: troubleshooting 5.

g_result <- gibbs_train( data=data,

 tree_str=tree_str,

 Phi=ge_result$Phi_samples,

 I=ge_result$I_samples,

 rho=ge_result$rho_samples,

 Z=ge_result$Z_samples,

 alpha=ge_result$alpha_samples,

 burn_in=2500, cycle=5000, interval=50 )

***Note:*** In addition to the arguments used in the “*gibbs_EM_train*” function, the “*burn_in*” argument sets the number of burn-in iterations of the Gibbs sampler; the “*cycle*” argument sets the total number of Gibbs sampling iterations; the “*interval*” argument sets the total number of Gibbs iterations to be run for one posterior sample of hidden variables to be collected.

***Note:*** Effective sample sizes of hidden variables can be calculated to assess the mixing of the Gibbs sampler (see step 7).

5. Inspect the result object:
  a. Check the inference result saved in the R object “*g_result*”.

**Figure 6:**
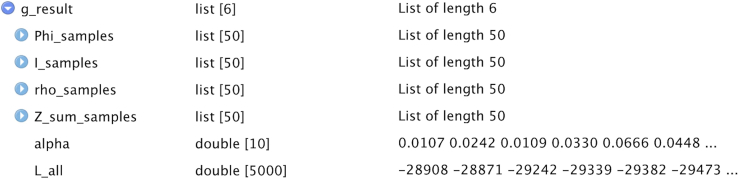
Elements in the result object given by the Gibbs sampler for the treeFLA model

***Note:*** The result object (“*g_result*”) given by Gibbs sampling stores posterior samples of hidden variables, intermediate variables, hyperparameters and summary statistics of treeLFA (Figure 6; Table 2).

**Pause Point:** Most of the inferred topics are expected to be distinct probability vectors with a limited number of diseases having large probability while other diseases having very small probabilities. The ranges and features of other variables can be found in Table 2 for a sanity check.

**Pause Point:** The “range” in Table 2 denotes the ranges of individual elements of the corresponding hidden variables.

***Note:*** “*Z_sum_samples*” is an intermediate variable created to enable the implementation of the inference of treeLFA (Table 2).

***Note:*** Unlike the “*ge_result*” object (given by the Gibbs-EM algorithm), the posterior samples of the topic assignment variables (“**Z**”) are not kept in the “*g_result*” object, as they are less useful than “*Z_sum_samples*”, which can be used to approximate peoples’ topic weights (see below).

***Note:*** The posterior samples of the variables “*Z_sum*” in the “*g_result*” object can occupy a relatively large memory space if a large number of posterior samples are kept. Each element of the “*Z_sum_samples*” list is a “number of individuals” by “number of topics” matrix. For users having a limited memory space, it is recommended to roughly estimate the overall memory requirement of implementing treeLFA using the space occupied by the two largest matrices (of equal size) kept during the inference (the input data matrix and the topic assignment matrix) and the space occupied by “Z_sum_samples”: $M\left(KB\right)=8*D*K*N_{ps}+8*D*S*2$, where “*M*” is the memory size in kilobyte, “*D*” is the total number of individuals, “*K*” is the total number of topics, “*N*_*ps*_” is the total number of posterior samples, and “*S*” is the total number of disease codes. The memory space occupied by other variables are orders of magnitude lower than them.

***Note:*** The topic weight variables of the treeLFA model (“**Theta**”) (see Figure 1 of the primary publication) are integrated out in the inference process, therefore they are not directly available from the result object at this stage. However, topic weights can be approximated using “*Z_sum_samples*”. In the post-processing steps, estimated topic weights for individuals can be obtained (see sections below).

***Note:*** Entries in “**alpha**” have a minimum value of 0.01, as we put a restriction on its lower bound to avoid degeneration. Apart from **“alpha”**, for other hyperparameters and hidden variables of treeLFA, conjugate priors are used (for instance, Beta prior and Bernoulli distribution for the probability variables of disease codes in topics, Dirichlet prior and categorical distribution for individuals’ topic weight vectors), such that their posterior distribution is analytically tractable.

**Table 2:** Variables of treeLFA model in the result object given by the Gibbs sampler

Name	Type	Range	Meaning	Format	Description
*Phi_samples*	Hidden variable	(0,1)	Probability of disease codes in topics	List	Each posterior sample of “**Phi**” is a probability matrix in the list. Each row of the matrix corresponds to a topic and each column corresponds to a disease code.
*I_samples*	Hidden variable	0 or 1	Indicators of disease codes in topics	List	Each posterior sample of “**I**” is a binary matrix, with one and zero indicating active and inactive disease codes in topics. Each row of the matrix corresponds to a topic.
*rho_samples*	Hidden variable	(0,1)	Transition probabilities of the Markov process on the tree structure of disease codes	List	Each posterior sample of “**rho**” is a vector of two probability, denoting the probability of an inactive/active disease code having an inactive child disease code on the tree structure, as the Markov process of generating indicators of diseases in a topic runs from the top (root node) to the bottom (terminal nodes) of the tree.
*Z_sum_samples*	Hidden variable	0,1,2,3…S^a^	Numbers of different topics assigned to all the disease codes for individuals	List	Each posterior sample is a count matrix. Each row of the matrix corresponds to an individual and each column a topic. Each entry denotes the total number of disease variables of an individual assigned with a specific topic.
*alpha*	Hyper-parameter	[0.01,+ ∞)	Dirichlet prior for individuals’ topic weights	Vector	Each entry corresponds to the weight of a specific topic.
*L_all*	Summary statistic	(-∞,0)	log10-likelihood of the treeLFA model	Vector	At each Gibbs sampling step, the log-10 likelihood of the model is calculated. The traceplot should show random, rapid fluctuation around a stable mean.

a S is the total number of disease codes analyzed.

### Post-processing of the inference result

**Timing: 1 h**

**Timing: 10–20 min (for step 6)**

**Timing: 10–20 min (for step 6a)**

**Timing: 10–20 min (for step 6b)**

**Timing: 10 min (for step 7)**

**Timing: Variable (for step 8)**

In this step, hyperparameters and posterior samples of hidden variables are processed (clustered and combined) to generate a single set of topics and topic weights ready for the downstream analyses.

**Figure 7:**
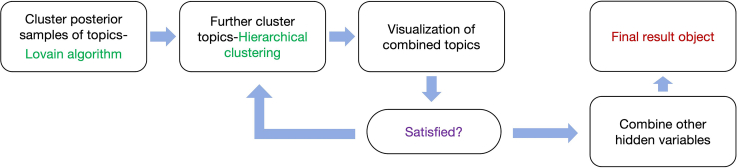
Schematic of major steps in post-processing the inference result for the treeLFA model

***Note:*** In this section we introduce the strategy we used in the primary publication. When analyzing real-world dataset, we do not know in advance the optimal number of topics to be inferred. The standard way of dealing with this problem is to train multiple models set with different numbers of topics, and then compare these models using various evaluation metrics (for instance, the predictive likelihood of the model on testing datasets, the coherence and diversity of the inferred topics^1^). Another method is to compare the results of downstream analyses for different models, checking if they make biological sense). This strategy is computationally expensive, as many models need to be trained and evaluated. Our strategy is to train only one treeLFA model with a sufficiently large number of topics. As a result of the excessive number of topics set for the model, there will be duplicated topics inferred. We cluster the inferred topics and combine duplicated ones, and then combine other hidden variables (e.g. people’s topic weights) accordingly. In this way, we extract strong patterns of disease co-occurrence in the data without training multiple models. Meanwhile, we also use multiple posterior samples of hidden variables to account for the uncertainty in estimation. The major steps in post-processing the inference result are shown in Figure 7.

***Note:*** The code to calculate the metrics of topics (topic coherence and topic diversity) is shown in sub-step c of step 6 in this section.

***Note:*** Also see the “post-processing of the inference result” subsection in the “Method Details” section for relevant details and Figure 5 for the rationale behind this strategy in the primary publication.

**Figure 8:**
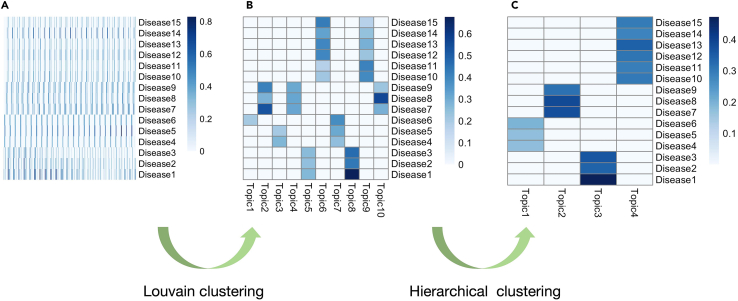
The two-steps clustering of posterior samples of topics (A) All posterior samples of all (ten) topics given by a Gibbs sampler. (B) The averaged topics after Louvain clustering of all posterior samples of topics. (C) The remaining distinct topics after combining similar topics using the Hierarchical clustering.

6. Cluster posterior samples of topics.
  Cluster all posterior samples of all topics in the result object given by the Gibbs sampler.
  ***Note:*** The result object given by the Gibbs sampler in the previous step contains multiple posterior samples of all hidden variables, and the order of the inferred topics in different posterior samples can be different, therefore topics from different posterior samples cannot be combined directly (the “identifiability” issue). In this step we cluster all posterior samples of all topics, such that corresponding topics in different posterior samples can be put into the same cluster and then combined.
  a. Louvain clustering of posterior samples of topics:
    i. Use the Louvain algorithm to cluster all posterior samples of all topics in the “*g_result*” object:
      g_result <- cls_louvain(g_result, k=25)
      ***Note:*** The “*g_result*” object in the above function is the result object given by the Gibbs sampling function for treeLFA in the previous step, it will be augmented in the following steps by the clustering functions via attaching new (clustering) results to it.
      ***Note:*** “*k*” is a parameter of the Louvain algorithm, which specifies the number of neighbors considered in building the shared nearest neighbor (SNN) graph during the clustering process. Large values for “*k*” will result in fewer clusters (as similar small clusters will be combined into a larger one), while small values for “*k*” will result in many smaller clusters (possibly duplicated clusters). Based on our experience of model training, we empirically recommend users to use an integer within the range of (“*N_ps/2*”, “*N_ps*”) for “*k*”, where “*N_ps*” is the total number of posterior samples of hidden variables given by the Gibbs sampler. Users can also try other values of “k” and decide which one give rise to the best clustering result (topics can be evaluated using the metrics introduced below).
      ***Note:*** Different sets of topics resulted from different value of “k” can be evaluated by metrics such as “topic coherence” (which measures if the top disease codes in a topic tend to co-occur on the same individuals in the input data) and “topic diversity” (which measures if the top disease codes in all topics cover a large enough proportion of all disease codes in the input data) (see more details and the relevant papers in the “evaluation of inferred topics” subsection of the “Method details” section in the primary publication).
      ***Note:*** Warning messages about tied distances to neighbors can be ignored.
    ii. Plot the averaged topic vector of each topic cluster (Figure 8A and 8B):
      topics_plot(g_result$topics_ave)
      ***Note:*** In the new "*g_result*" object, the "*topics_ave*" matrix stores the averaged topic vector for each cluster.
      ***Note:*** As can be seen in Figure 8B, after the Louvain clustering there are still ten clusters of posterior samples of topics, and there are apparently “duplicated” topics (refer to the topics with similar structure yet slight differences in the probabilities of active disease codes, such as topics 6 and 9 in Figure 8B). This is within expectation since the Louvain algorithm alone is not enough to combine slightly different topic clusters. In the next step, hierarchical clustering will be applied to combine duplicated topics such that the final set of distinct topics can be obtained.
      ***Note:*** If treeLFA is repetitively run for multiple times, the inference result can be slightly different. Here we only use one instance of inference result as an example to illustrate the whole analytic process. The reason of different inference result given by different chains is that treeLFA model contains a large number of hidden variables, and on real world data it is not possible to fully explore the joint posterior distribution of all hidden variables with limited computing resource, therefore chains can be stuck in different local optimum of the posterior distribution. A practical strategy of dealing with this issue is to train multiple treeLFA chains, and focus on topics that are repetitively inferred by different chains (see the “Analyses on hundreds of ICD10 codes in UKB” subsection in the “Results” section of the primary publication).
  b. Hierarchical clustering of topic clusters:
    Further combine very similar topics from the previous step by applying hierarchical clustering algorithm.
    g_result <- cls_hier(g_result, k=4)
    ***Note:*** The hierarchical clustering algorithm will combine the pair of most similar topics among all pairs of topics each time, until the requested number of topics is obtained.
    ***Note:*** The “*k*” parameter in the “*cls_hier*” function specifies the number of distinct topics to retain. The default and preferred distance metric used for the hierarchical clustering is the “cosine distance”, which is not sensitive to the small differences between the corresponding elements in similar topic vectors. From Figure 8B we know that there are roughly four distinct topics, so we set “*k*” to be four here.
    ***Note:*** Topic 1 in Figure 8B only has one active code (disease code with a probability much larger than zero). It is not a meaningful topic, since topics should reflect groups of diseases that tend to co-occur. With topic 1 excluded, there are about four distinct topics.
    ***Note:*** treeLFA tends to infer empty topic(s), which means a topic with no active codes, when an excessive number of topics are set for the model. In theory, people’s weights for the empty topic reflect their disease burden (the total number of diseases diagnosed) and the multimorbidity burden (the total number of topics that the diagnosed diseases come from). Interestingly, we found that the empty topic had many unique genomic associations (see paragraph 6 in the “Discussion” section of the primary publication). After clustering of topics, one empty topic can be retained, which means for the example dataset there will be five topics remained at last: four topics corresponding to the four true topics used to simulate the input data, and one empty topic. The empty topic will not interfere with the interpretation and downstream analyses of the non-empty disease topics. Note that empty topic(s) will always be inferred on real-world dataset.
    ***Note:*** Besides applying the hierarchical clustering, using other algorithms (such as k-means and spectral clustering) after the Louvain clustering of topic vectors should also work. Extensive comparisons between these algorithms were not made, as on real-world dataset the number of the averaged topic vectors remained after using the Louvain algorithm will be small, and most of them will be distinct topics except for those similar “near-empty” topics. In this case, the hierarchical clustering is the simplest and most straightforward solution to further combining similar topics.
    ***Optional:*** Visualize the combined topics after the hierarchical clustering.
    topics_plot(g_result$topics_ave_h)
    ***Note:*** In the updated “*g_result*” object, the “*topics_ave_h*” matrix stores the combined topics kept after the hierarchical clustering.
    ***Note:*** This time four distinct topics are obtained (Figure 8C), which look very similar to the four true topics (Figure 3) used to simulate the input data.
    ***Note:*** The hierarchical clustering can be done multiple times (gradually decreasing the number of topics kept) until a satisfactory set of topics are obtained.
    ***Optional:*** Evaluate the inferred topics.
  c. Calculate “topic coherence” and/or “topic diversity” of topics to evaluate the inferred topics:
    topics_coherence(topics=g_result$topics_ave_h, data=data)
    topics_diversity(topics=g_result$topics_ave_h, data=data, N=N)
    ***Note:*** For the parameters set in the above two functions, “*data*” is the input data for the treeLFA model, and “*N*” in the “*topics_diversity*” function specifies the number of top disease codes to be considered.
    ***Note:*** There are various metrics that can be used to evaluate the inferred topics from different perspectives. Two of the commonly used ones are “topics coherence” and “topic diversity”.
    “Topic coherence” measures the tendency of top disease codes in topics to co-occur on the same individuals in the training dataset. It is calculated as follow:
    $$TC=\frac{1}{K}\sum_{k=1}^{K}\frac{1}{S\left(S-1\right)}\sum_{i=1}^{S-1}\sum_{j=i+1}^{S}f\left(c_{i}^{k},c_{j}^{k}\right)\text{,}$$
    “TC” is the topic coherence, “K” is the total number of topics, “S” is the total number of disease codes, ${{\prime \prime c}_{i}^{k}}^{\prime \prime }$ is the i^th^ disease code in topic k, and “f(.,.)” is the normalized pointwise mutual information of two disease codes:
    $$f\left(c_{i},c_{j}\right)=\frac{\log\left(\frac{P\left(c_{i},c_{j}\right)}{P\left(c_{i}\right)\cdot P\left(c_{j}\right)}\right)}{-\log P\left(c_{i},c_{j}\right)}\text{,}$$
    in the above equation, “$P\left(c_{i}\right)$” is the marginal probability of disease code i in the training data, and “$P\left(c_{i},c_{j}\right)$” is the probability of disease codes i and j co-occur on the same individuals (approximated using the empirical counts in the training data).
    “Topic diversity” measures the number of unique disease codes covered by the top N disease codes in all the inferred topics:
    $$\text{TD}=\frac{U}{N\cdot K}\text{,}$$
    *“U”* is the total number of unique disease codes among the top “*N*” disease codes in all the “*K*” topics.
7. Clustering of hidden variables other than topics:
  a. Cluster other hidden variables of treeLFA:

g_result <- cls_others(g_result)

***Note:*** In the previous steps, only posterior samples of topics are clustered, as topics are the most important variables of treeLFA. Besides, topics are easy to visualize, distinguish and process. After the total number of distinct topics to keep is determined, hyperparameters and the posterior samples of other hidden variables can be put into the corresponding clusters (according to the clustering result of topics) and then averaged.

***Note:*** In each posterior sample of hidden variables, the entries of the hyperparameter vector “**alpha**”, topics and peoples’ weights for topics have one to one correspondence. This means that once the cluster assignments of topics are determined, other hidden variables can then be directly put into the corresponding clusters.

***Note:*** In the new “*g_result*” object, the “*tw_ave*” matrix stores the averaged combined topic weights of all individuals, and the “*alpha_ave*” is the averaged combined hyperparameter “**alpha**”.

***Optional:*** Plot individuals’ topic weights.

tw_plot(g_result$tw_ave[1:5,])

**Figure 9:**
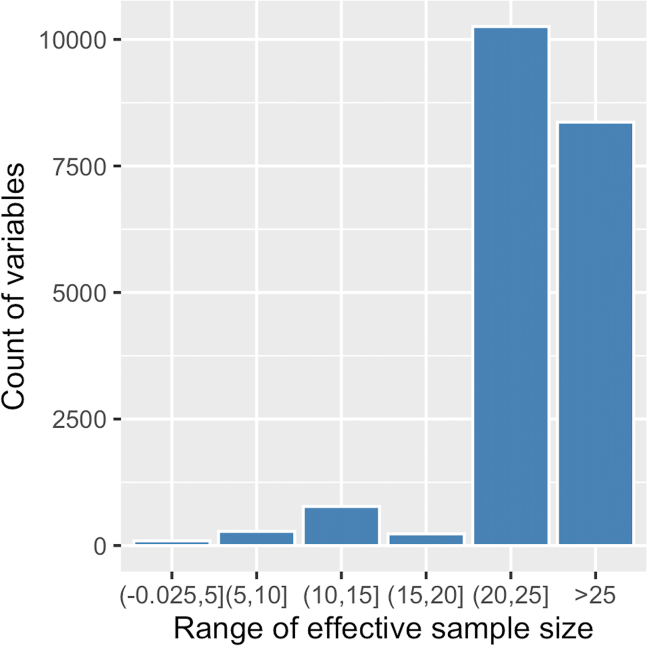
Distribution of effective sample sizes for all topic weight variables

***Note:*** The first five individuals' topic weights are plotted for visualization (Figure 9).

***Optional:*** Plot the effective sample sizes of the posterior samples of hidden variables.

ess_plot(g_result$ess_tw, bin_N=5, N_ps=25)

**Figure 10:**
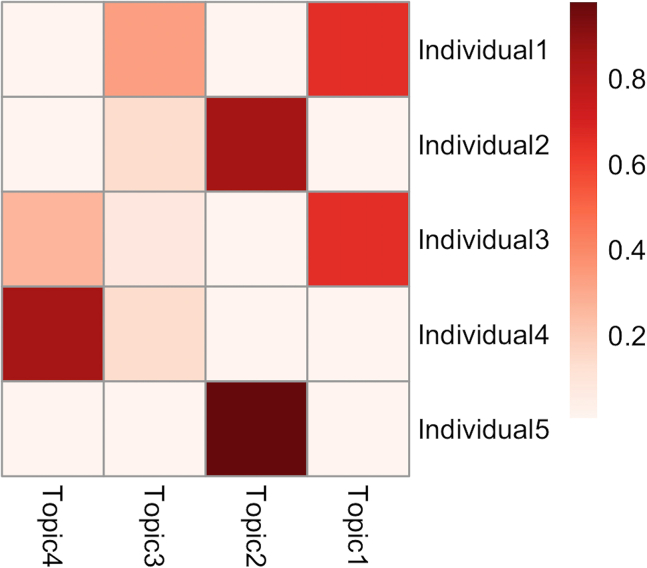
Averaged topic weights for the first five individuals in the example input dataset

***Note:*** The effective sample size (ess) of hidden variables can be calculated to examine the mixing of Gibbs sampling. In the “*cls_others*” function, the ess of all disease probability variables in topics (4 × 15 variables in total) and topic weight variables (5000× 4 variables in total) are calculated, and stored in the “*ess_topics*” and “*ess_tw*” lists in the “*g_result*” object. The distribution of ess of hidden variables can be plotted using the “*ess_plot*” function (“*bin_N*” is the number of bins set for ess, and “*N_ps*” is the number of posterior samples collected by the Gibbs sampler) (Figure 10).

***Note:*** The “topics_all” and “tw_all” lists in the “*g_result*” object stored posterior samples of different topics/topic weights separately (for example, the first element, a 15 × N_ps matrix in the “topics_all” list stores the “N_ps” posterior samples of the first topic vector of length 15). They can be extracted and used for further analyses and plotting (for instance, the distribution of people’s weights for a specific topic).

8. Downstream analyses:
  a. Carry out the downstream analyses (such as topic-GWAS) using the inference result generated in the previous steps.

***Note:*** With topics of diseases and people’s topic weights, downstream analyses can be carried out. One of the analyses can be done is “topic-GWAS”, which means using people’s inferred topic weights as continuous traits in GWAS. The input for topic-GWAS is peoples’ inferred topic weights for different topics and peoples’ genotype data. Notably, people’s topic weights always fall into the range of zero to one, meaning they do not follow Gaussian distribution. Therefore, standard linear regression may not be the suitable model to fit. To solve this issue, a logit transformation on topic weights is suggested. Details can be seen in the primary publication (Figure 5 and the “Genetic analyses” subsection in the “Method Details” section).

***Note:*** For topic-GWAS, it is still recommended to include common covariates in the analysis, including age, sex and the principal components of genetic variations of individuals. The exclusion of certain diseases is not necessary.

***Note:*** For the multiple testing correction issue, if specific topics are independently the traits of interests for topic-GWAS, then multiple testing correction is not necessary for the total number of topics. However, if researchers are interested in the effect of certain SNPs across multiple disease topics, it is still recommended to correct for both the number of SNPs and number of topics.

## Expected outcomes

All inference results are stored in the “*g_result*” object, which can be saved as an RData object and loaded into the RStudio again. The most important inference result for treeLFA are the topics of disease codes and people’s topic weights for topics, which are two averaged matrices store in the “*g_reuslt*” object (“*topics_ave_h*” and “*tw_ave*”). They can be used directly in the downstream analyses.

## Limitations

The main limitation of treeLFA is the long computational time of performing inference. The total computational time is linearly proportional to the total number of disease codes analyzed and the total number of individuals in the training dataset. On large biobank datasets, the Gibbs-EM training can take up to weeks (depending on the properties of the computing facility and the exact size of the input data). In the future, other optimization algorithms may further speed up the inference.

## Troubleshooting

### Problem 1

• The installation and loading of R packages and the sourcing of R scripts are not successful.
• There are required packages (for instance, the “scran” package requires the “edgeR” package, the “pheatmap” package requires the “colorspace” package. These packages may need to be installed manually) that have not been installed and loaded.
• On macOS operating system, the compilation of C++ code needs the “Xcode” software, but its license agreements have not be agreed by the user. On windows operating system, the “Rtools” software is required to source C++ code but have not been installed.

### Potential solution

• Install the required R packages according to the error messages.
• Run commands to agree to the “Xcode” agreements or install “Rtools” as instructed by the error messages.

### Problem 2

• The inference of treeLFA cannot be performed due to formatting issues of the input diagnosis data matrix (related to Step 1):
• The input matrix is not a binary integer matrix;

### Potential solution

Prepare the input diagnosis data as instructed in Step 1.

***Note:*** An error message will appear in the situations listed above while running the Gibbs-EM algorithm.

### Problem 3

• The inference of treeLFA cannot be performed due to formatting issues of the tree structure matrix (related to Step 2).
• The tree structure of disease codes (the 1^st^ column of the tree structure input matrix) and the input diagnosis data matrix (the column names of the matrix) have different disease codes;
• The tree structure matrix contains repetitive disease codes in the 1^st^ column;
• The 1^st^ column of the tree structure matrix contains the “root” code or there is no “root” code in the 2^nd^ column of the tree structure matrix;
• The parent codes in the 2^nd^ column of the tree structure matrix have overlaps with the disease codes in the input diagnosis data matrix (should be terminal disease codes on the tree structure);
• The parent codes in the 2^nd^ column of the tree structure matrix (except for the root code) do not appear in the 1^st^ column of the tree structure matrix.
• The parent codes in the 1^st^ column of the tree structure matrix are not in the 2^nd^ column of the tree structure matrix.

***Note:*** The sets of parent codes in the 1^st^ and 2^nd^ column of the tree structure matrix should be exactly the same. The algorithm will check this.

### Potential solution

Prepare the tree structure matrix as instructed in Step 2.

***Note:*** An error message will appear in the situations listed above.

### Problem 4

• The inference of treeLFA cannot be performed due to wrong parameters passed to the function.
• The number of topics passed to the function does not equal the length of the “**alpha**” vector;

### Potential solution

Set the parameters correctly (Refer to step 3 in the “perform inference of treeLFA model” section).

***Note:*** An error message will appear in the situations listed above while running the Gibbs-EM algorithm.

### Problem 5

• The posterior samples of treeLFA cannot be simulated due to wrong parameters passed to the function.
• The value set for the “*burn_in*” parameter should be smaller than the value set for the “*cycle*” parameter, and the value for the “interval” parameter should be smaller than the value set for “*cycle*” minus “*burn_in*”.

### Potential solution

Set the parameters correctly (Refer to step 4 in the “perform inference of treeLFA model” section).

***Note:*** An error message will appear in the situations listed above while running the Gibbs sampling algorithm.

### Problem 6

• The mixing/convergence of Gibbs chains is not satisfactory.
• The Gibbs chain is stuck in the local optimum of the multi-modal posterior distribution of hidden variables.
• The initialization values of hidden variables lie in the low probability region of their posterior distribution.
• The choice of hyperparameters is not appropriate.
• The input data is very sparse, resulting in large variance of the posterior distribution of certain hidden variables.

### Potential solution

• In theory, taking more samples from the Gibbs chains can result in a more accurate estimation of the posterior distribution of hidden variables. But in reality, there is only limited computational resource and training time. In addition, the input data for treeLFA is sparse, and the treeLFA model has an “identifiability” issue for topics. These factors can all slow down the convergence/mixing of Gibbs-EM/Gibbs chains. Therefore, simply prolonging the training may not be enough.
• A practical way of dealing with this problem is to train multiple Gibbs-EM/Gibbs chains with different initialization of hyperparameters and hidden variables (for instance, try random initialization for the indicator variables “**I**” of disease codes in topics, different Beta priors for the transition probability variable “**Rho**”, and a different initialization strategy for “**alpha**”). Chains with poor convergence/mixing (identified using the diagnostic metrics introduced in the previous sections) can be discarded, and then the inference result of the remaining chains can be compared and used.
• Users can try to train treeLFA models with a different number of topics, possibly after some exploratory analyses which shed light on the optimal number of topics to set for the model.
• Users can try to change the values of the parameters of treeLFA training (for instance, different values for parameters “opt_N_1”, “opt_N_2” and “burn_in” of the Gibbs-EM training).
• If the traceplot shows that the full likelihood of the model is still having an increasing trend, users can prolong the training moderately.

## Resource availability

### Lead contact

Further information and requests for resources and reagents should be directed to and will be fulfilled by the lead contact, Gerton Lunter (g.a.lunter@umcg.nl).

### Technical contact

Questions about the technical specifics of performing the protocol should be directed to the technical contact, Yidong Zhang (zhangyidong@pumch.cn).

### Materials availability

This study did not generate new unique reagents.

### Data and code availability

• A RMarkdown demonstration for using the algorithm on a small, simulated example dataset is available at: https://zhangyd10.github.io/treeLFA-demo-CG or https://doi.org/10.5281/zenodo.8077452.
• Code for the treeLDA algorithms is available at: https://github.com/zhangyd10/treeLFA-demo-CG.

## Acknowledgments

This work was supported by the 10.13039/501100005150Chinese Academy of Medical Sciences (CAMS) Innovation Fund for Medical Science (CIFMS), China (grant number: 2018-I2M-2-002). The research was supported by the 10.13039/100010269Wellcome Trust Core Award grant number 203141/Z/16/Z with additional support from the 10.13039/100014461NIHR Oxford BRC and the Wellcome Trust Award grant number 100956/Z/13/Z.

## Author contributions

Y.Z.: conceptualization, data curation, formal analysis, methodology, software, validation, visualization, and writing – original draft; X.J.: conceptualization, data curation, methodology, software, and writing – review and editing; G.L.: conceptualization, investigation, methodology, project administration, resources, supervision, writing – original draft, and writing – review and editing; G.M.: conceptualization, funding acquisition, methodology, project administration, resources, supervision, and writing – review and editing.

## Declaration of interests

G.M. is a director of and shareholder in Genomics PLC and a partner in Peptide Groove LLP. G.L. is a shareholder in Genomics PLC.
